# Deficiency of Dietary Fiber in *Slc5a8*-Null Mice Promotes Bacterial Dysbiosis and Alters Colonic Epithelial Transcriptome towards Proinflammatory Milieu

**DOI:** 10.1155/2019/2543082

**Published:** 2019-12-27

**Authors:** Sathish Sivaprakasam, Pramodh K. Ganapathy, Mohd Omar Faruk Sikder, Moamen Elmassry, Sabarish Ramachandran, Kameswara Rao Kottapalli, Vadivel Ganapathy

**Affiliations:** ^1^Department of Cell Biology and Biochemistry, Texas Tech University Health Sciences Center, Lubbock, TX 79430, USA; ^2^Washington University School of Medicine, St. Louis, MO, USA; ^3^Department of Biological Sciences, Texas Tech University, Lubbock, TX 79409, USA; ^4^Department of Biotechnology, Genomic Center, Texas Tech University, Lubbock, TX 79409, USA

## Abstract

Inflammatory bowel disease (IBD) is characterized by chronic inflammation in the intestinal tract due to disruption of the symbiotic relationship between the host immune system and microbiota. Various factors alter the gut microbiota which lead to dysbiosis; in particular, diet and dietary fibers constitute important determinants. Dietary fiber protects against IBD; bacteria ferment these dietary fibers in colon and generate short-chain fatty acids (SCFAs), which mediate the anti-inflammatory actions of dietary fibers. SLC5A8 is a high-affinity transporter in the apical membrane of colonic epithelium which mediates the entry of SCFAs from the lumen into cells in Na^+^-coupled manner. Due to the unique transport kinetics, the function of the transporter becomes important only under conditions of low dietary fiber intake. Here, we have examined the impact of dietary fiber deficiency on luminal microbial composition and transcriptomic profile in colonic epithelium in wild-type (WT) and *Slc5a8*-null (KO) mice. We fed WT and KO mice with fiber-containing diet (FC-diet) or fiber-free diet (FF-diet) and analyzed the luminal bacterial composition by sequencing 16S rRNA gene in feces. Interestingly, results showed significant differences in the microbial community depending on dietary fiber content and on the presence or absence of Slc5a8. There were also marked differences in the transcriptomic profile of the colonic epithelium depending on the dietary fiber content and on the presence or absence of Slc5a8. We conclude that absence of fiber in diet in KO mice causes bacterial dysbiosis and alters gene expression in the colon that is conducive for inflammation.

## 1. Introduction

Inflammatory Bowel Diseases (IBD) including Crohn's disease and ulcerative colitis are characterized by chronic inflammation in the intestine. IBD is a complex disease with multiple etiological factors involved in its development and pathogenesis. Among them, environmental factors such as diet and the microbiome play a critical role [1, 2]. Another important factor is epigenetic modifications, which plays a vital role in the proper functioning and maintenance of intestine by controlling the development of the intestinal epithelium and the immune cells in the lamina propria [3]. The intestinal tract is in continuous intimate contact with microbiota. Under normal physiological conditions, both the epithelial cells as well as the immune cells in the lamina propria contribute to the maintenance of the intestinal barrier function and to the development of tolerance to the bacteria found in the normal colon. Conditions that disrupt the functions of the epithelial cells and the immune cells alter the composition of the bacteria in the colon; such changes in colonic bacteria, known as dysbiosis, are well recognized as important etiological factors in the pathogenesis of IBD [4, 5].

Previous studies have shown that diet is a vital factor in shaping the gut microbiota and changes in the dietary components profoundly alter microbial communities, thereby increasing the susceptibility to various diseases [4, 5]. The western diet is significantly deficient in fiber, much lower than the normal recommended value [6]. Diet deficient in fiber causes dysbiosis that leads to breakdown of the epithelial barrier function and activation of the immune system leading to proinflammatory conditions [5]. In contrast, diet rich in fiber enhances epithelial barrier function, suppresses immune function, and protects against immune activation [7]. In addition to these beneficial effects on the host, dietary fiber also provides energy substrates for gut bacteria and determines the relative abundance of various bacterial strains that reside in the gut. When the diet is deficient in fiber, gut microbiota uses the carbohydrates present in the mucus layer of the gut as energy substrates, consequently thinning the protective mucus layer [7].

SLC5A8 is a Na^+^-coupled high-affinity transporter for SCFAs; it is located in the lumen-facing apical membrane of colonic epithelial cells [8–10]. SLC5A8 is a candidate tumor suppressor, whose expression is silenced in colon cancer [11–13]. SCFAs are effective inhibitors of histone deacetylases (HDACs), but this process depends on how effectively these SCFAs enter the epithelial cells [14]. As SLC5A8 is a high-affinity transporter, its contribution to the cellular entry of SCFAs is negligible when the luminal concentrations of SCFAs are in millimolar range as occurs when dietary fiber intake is optimal. Under these conditions, low-affinity transporters for SCFAs such as the monocarboxylate transporter MCT1 (SLC16A1) are primarily responsible for the entry of SCFAs into colonic epithelium [13]. Luminal concentrations of SCFAs are decreased when the dietary fiber intake is low; under these conditions, the high-affinity transporter SLC5A8 becomes important for the entry of SCFAs into colonic epithelium [13]. This has been demonstrated convincingly using *Slc5a8*-null mice. With optimal fiber content in diet, there is no difference between wild-type mice and *Slc5a8*-null mice in the outcome of experimentally induced colitis; but when the dietary fiber content is low, *Slc5a8*-null mice show increased disease severity in experimentally induced colitis [15].

Previous reports from our lab have shown that microbiota influences the expression of Slc5a8 in colon; germ-free mice have markedly reduced expression of Slc5a8, and recolonization of the colon with bacteria increases Slc5a8 expression [9]. Similarly, enhancement of colonic bacteria with the exogenous administration of probiotic strains in mice enhances Slc5a8 expression [16, 17]. In the present study, we investigated the relationship between Slc5a8 and dietary fiber content in terms of the composition of colonic bacteria and colonic epithelial cell gene expression. The goal was to understand at the molecular level why *Slc5a8*-null mice are prone to colonic inflammation only under conditions of reduced fiber intake in the diet.

## 2. Materials and Methods

### 2.1. Animals

C57BL/6 mice (stock no. 000664) were obtained from Jackson laboratories. Generation of *Slc5a8*^−/−^ mice has been described [18], and mice were bred and maintained in Texas Tech University Health Sciences Center Laboratory Animal Resource Center (LARC) in accordance with the guidelines of the Institutional Animal Care Use Committees. Mice were maintained in the conventional animal housing with 12 h day-night cycles, with water and food provided ad libitum, and used between 8–12 weeks of age.

### 2.2. Animal Diets

Mice were fed a diet containing dietary fibers (fiber-containing diet or FC-diet) or a diet without fibers (fiber-free diet or FF-diet). These diets were custom-produced by Harlan Laboratories (Indianapolis, IN, USA). The diets were autoclaved and vacuum-packed by the manufacturer and kept at 4°C until they are used to feed the animals. The diets were provided to the animals ad libitum.

### 2.3. Feces Collection, Storage, and DNA Extraction

Each mouse was placed separately in a nonbedded cage for 4 h, and their fecal pellets were collected. The fecal pellets were stored at −80°C immediately and used for DNA isolation. Total DNA was isolated from fecal samples using the MoBio PowerSoil® DNA Isolation Kit (MoBio Laboratories, Inc., Carlsbad, CA) according to the manufacturer's instructions. The extracted DNA was stored at −80°C until library preparation and metagenomics sequencing were performed.

### 2.4. Library Preparation and 16S rRNA Gene Sequencing and Data Analysis

Library preparation and sequencing were performed at the Center for Biotechnology and Genomics, Texas Tech University, Lubbock TX using the Illumina 16S-metagenomics library prep protocol. Paired-end sequencing was performed on an MiSeq using a 600 cycle reagent cartridge. The forward and reverse adapters were trimmed, samples were demultiplexed, and fastq.gz files were generated using MiSeq reporter software (MSR) from Illumina. All the sequence files were uploaded into the NCBI-Sequence Read Archive (SRA) and Bio project ID: PRJNA515739 and used for data analysis. Sequencing files were used for further analysis with QIME (version 1.8.0), PEAR software, UCLUST algorithm and PyNast aligner software [19–22]. Texas Tech University high performance computational resource, Hrothgar was used to accomplish this computational data analysis.

### 2.5. Statistical Analysis of Sequencing Data

QIIME was used to calculate the species richness and diversity indices (Shannon, phylogenetic, and Chao1) in order to measure *α* diversity within the sample. Pairwise distances between microbial communities based on phylogenic relatedness of whole communities were calculated using UniFrac method (*β* diversity between samples) [23]. Indicator species analysis was performed to determine the indicative species of each group of samples using “indicspecies” function in R [24].

### 2.6. Total RNA Extraction

The colonic mucosal scrapings from WT and *Slc5a8*^−/−^ mice fed with the two different diets were collected. Total RNA was extracted using TRIzol reagent (Invitrogen Life Technologies, NY, USA) according to the manufacturer's instructions. Total RNA concentrations were quantified via Qubit® 3.0 Fluorometer and RNA HS assay kit (Thermo Fisher, MA, USA). Quality of RNA was checked using RNA ScreenTapes on Agilent 2200 TapStation (Santa Clara, CA, USA).

### 2.7. Library Preparations and RNA Sequencing

Total RNA was used for the cDNA library construction using TruSeq® Stranded mRNA LT kit (Illumina, San Diego, USA) and epMotion 5075t robot (Eppendorf, Hamburg, Germany). Library construction produced single-indexed libraries with a median insert size of ∼300 bp which was validated on an Agilent 2200 TapeStation instrument using D1000 ScreenTapes (Santa Clara, CA, USA). All libraries were quantified in triplicate using SynergyH1 fluorescent plate reader (BioTek, Vermont, USA). The pooled denatured cDNA libraries were loaded on a cBot for cluster generation followed by 2 × 108 bp paired-end sequencing using HiSeq Rapid kits with V2 chemistry on an HiSeq 2500 sequencer (Illumina, San Diego, USA). All the sequence files were uploaded into the NCBI-Sequence Read Archive (SRA) and Bio project ID: PRJNA517543 and used for data analysis.

### 2.8. Bioinformatics

The quality of the raw reads was assessed using FastQC software (Babraham Bioinformatics). Quality filtered reads (both reads 1 and 2) for each animal from each tissue sample were mapped to the mouse genome using QSeq® version 15.0 software (DNASTAR, Madison, WI, USA) for differential gene expression analysis using RPKM normalization. Differential gene expression analysis was performed by comparing grouped experimental samples to their corresponding grouped control samples. Genes were categorized as differentially expressed and statistically significant if they met 95% confidence (Student's *t*-test and the Benjamini–Hochberg false discovery rate method) and a cutoff of 2-fold change. Using standard setting with duplicates resolved, the gene list files were uploaded into Ingenuity Pathway Analysis (IPA) tool core analysis. IPA analysis comprised ascertaining canonical pathways, upstream regulators, and diseases and functions.

### 2.9. Western Blot

Colon mucosal scrapings were collected from wild-type and *Slc5a8*^−/−^ mice and homogenized in RIPA buffer (Thermo Scientific, USA) and supplemented with a proteases cocktail. Proteins were run on to SDS/PAGE gels and then transferred on to PVDF membranes. Membranes were blocked with bovine serum albumin, incubated with primary antibody at 4°C overnight, followed by treatment with appropriate secondary antibody conjugated to horseradish peroxidase (Bio-rad, USA). The antigen/antibody reaction was detected by the Enhanced Chemiluminescence Western blotting substrate (Thermo Scientific, USA). Primary antibodies were obtained from the following sources: p-Akt (cell signaling #4060), Akt (cell signaling #4691), HIF-1*α* (Novous #NB100-479), and *β*-actin (Santa Cruz #47778).

### 2.10. Statistical Analysis

The data shown are representative results of the means ± standard error of mean. Statistical significance was calculated using the Student's *t*-test with two-tailed analysis, unless stated otherwise. Differences were judged to be statistically significant when the *P* value was <0.05.

## 3. Results

### 3.1. Body Weight Change

**Figure 1:**
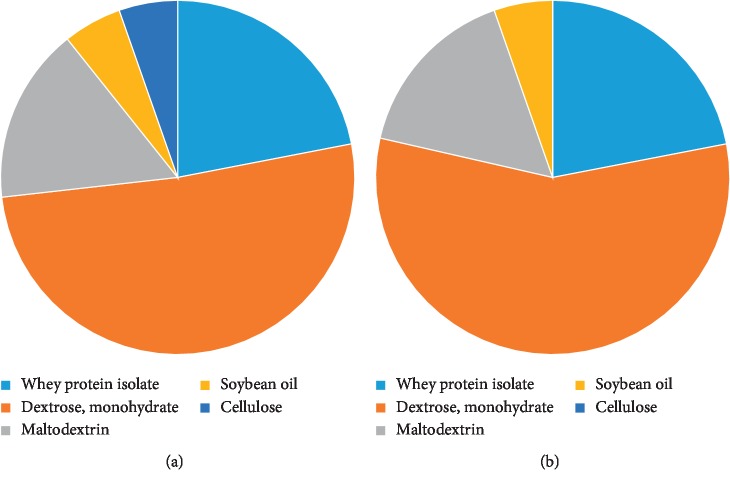
Components of (a) fiber-containing diet (FC-diet) and (b) fiber-free diet (FF-diet).

To understand the association between gut microbes and influence of dietary fiber with the function of Slc5a8, we fed age- and gender-matched wild-type and *Slc5a8*-null mice with fiber-containing diet (FC-diet) or fiber-free diet (FF-diet). The difference between FC-diet and FF-diet is the presence or absence of 5% cellulose, respectively, as a source of dietary fiber. The composition of the two diets is shown Figure 1. The diets were provided to the animals ad libitum. To evaluate the role of diet on mouse health, we monitored body weight over the entire period of the experiment; there was no significant difference in the body weight (data not shown). At the end of the experiment when the mice were sacrificed, we measured colon length, which alters under conditions of active inflammation; again, we did not see any significant difference among the different experimental groups (data not shown).

### 3.2. Microbial Diversity and Bacterial Abundance

**Figure 2:**
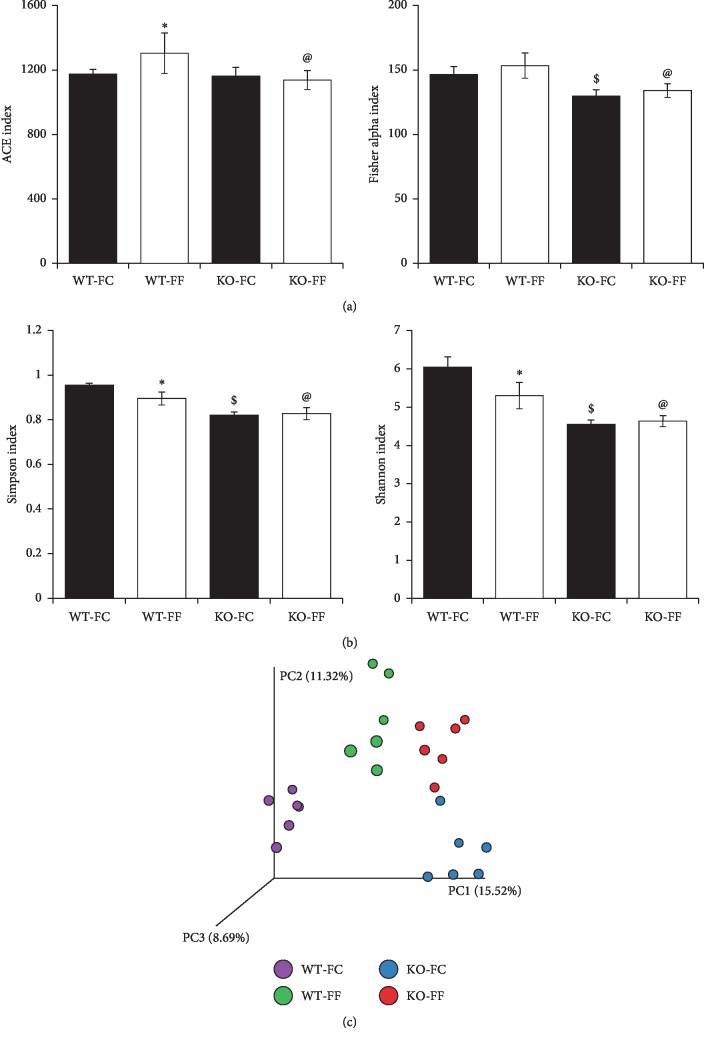
*α* and *β* diversities in fecal microbiota of WT and *Slc5a8*^−/−^ (KO) mice fed with fiber-containing diet (FC-diet) and fiber-free diet (FF-diet). Microbial richness was analyzed based on the ACE index and Fisher alpha index (a); Simpson index and Shannon index (b); unweighted Principal Coordinate Analysis-UniFrac metrics (c). The Student's two-tailed *t*-test was used to calculate statistical significance (*n* = 6 mice/group). ^*∗*^, *P* < 0.05 when compared between WT mice fed the FC-diet and WT mice fed the FF-diet; $, *P* < 0.05 when compared between WT mice fed the FC-diet and KO mice fed the FC-diet; @, *P* < 0.05 when compared between WT mice fed the FF-diet and KO mice fed the FF-diet.

To evaluate the influence of dietary fiber and the presence or absence of Slc5a8 on microbiota composition in colon, we collected feces from wild-type and *Slc5a8*^−/−^ mice fed either the FC-diet or the FF-diet. We performed sequencing of 16S rRNA gene using DNA samples isolated from these fecal samples. The richness and diversity of microbiota were assessed by alpha and beta diversity analysis (Figure 2(a)). We observed increased richness in wild-type mice fed the FF-diet when compared with wild-type mice fed the FC-diet. Such difference was not observed in *Slc5a8*-null mice when fed the two diets. More importantly, we found an interesting difference between wild-type mice and *Slc5a8*-null mice in bacterial richness when fed the FF-diet but not when fed the FC-diet. The richness was less in *Slc5a8*-null mice compared with wild-type mice. The observed changes in bacterial richness with regard to the two different diets and the two different genotypes of the mice were similar irrespective of whether the analysis was done using the ACE index or the Fisher's alpha index. We then analyzed the bacterial diversity among the four groups using two different methods (Simpson index and Shannon index) (Figure 2(b)). Decreased diversity of microbiota was observed in wild-type mice fed the FF-diet, null mice fed the FC-diet, and null mice fed the FF-diet compared with wild-type mice fed the FC-diet. More importantly, there was a decreased diversity in the null mice than in the wild-type mice irrespective of the fiber content in the diet. Interestingly, the absence of fiber in the diet decreased the bacterial diversity in the wild-type mice, but this was not the case in the null mice. The bacterial diversity remained the same in the null mice irrespective of whether or not the diet contained fiber.

To compare the microbiome community structure of the fecal samples across groups regarding their phylogeny, we used three-dimensional Principal Coordinate Analysis (PCoA) of unweighted UniFrac distances (which considers only OTU presence and absence, Figure 2(c)). The microbiota samples neatly fell into four clusters based on the mouse genotype and dietary fiber condition. These results suggest that there is a difference in the microbiota community with dietary fiber content, and that Slc5a8 genotype (i.e., presence or absence) induces further changes in the microbiota.

**Figure 3:**
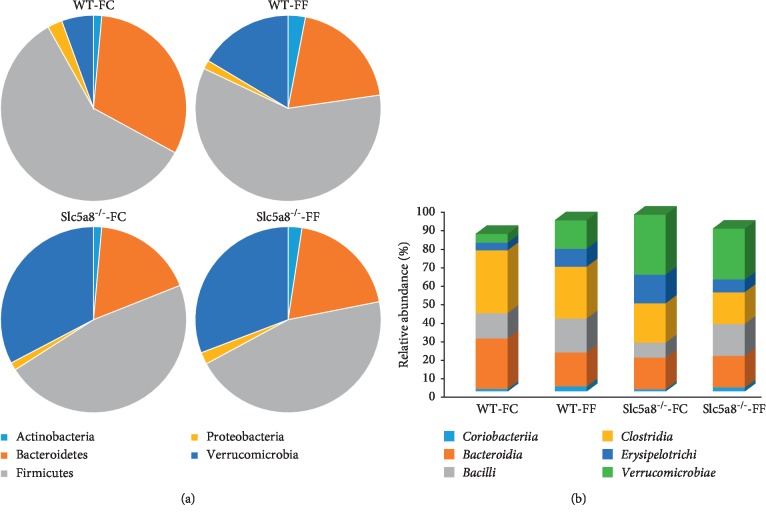
Taxonomic level difference in the phylum and class level. Phylum and class level abundance is expressed as % of fecal microbiota in the experimental group. Data represent only the predominant phyla and the class whose abundance shows the most significant difference (*n* = 6).

We then assessed the role of dietary fiber and the Slc5a8 genotype on the relative abundance of microbiota at different taxonomic levels. Decreased abundance of *Bacteroidetes* was observed in wild-type mice fed the FF-diet and in Slc5a8-null mice fed either the FC-diet or the FF-diet compared with wild-type mice fed the FC-diet (Figure 3(a)). A similar trend was observed in the abundance of *Firmicutes*. The presence or absence of fiber in the diet also altered the abundance of *Verrucomicrobia* significantly. Our phylum analysis clearly showed that *Verrucomicrobia* abundance was inversely proportional to dietary fiber content; the abundance was greater in wild-type mice fed the FF-diet than in wild-type mice fed the FC-diet. More importantly, the presence or absence of Slc5a8 impacted specifically on the abundance of this phylum. There was an increased abundance of *Verrucomicrobia* in the null mice irrespective of the presence or absence of fiber in the diet compared with wild-type mice when fed the corresponding diet.

At the class level, FF-diet increased in both wild-type mice and in *Slc5a8*-null mice, the abundance of *Coriobacteriia*, which belongs to the phylum *Actinobacteria* and *Bacilli*, which belongs to the phylum *Firmicutes* when compared with wild-type mice fed the FC-diet (Figure 3(b)). Interestingly, *Slc5a8*^−/−^ mice fed the FF-diet also showed significantly increased *Coriobacteriia* when compared with *Slc5a8*^−/−^ mice fed the FC-diet (Figure 3(b)). The abundance of *Clostridia*, another class within the *Firmicutes* phylum, showed a decrease in the feces of mice fed the FF-diet when compared with mice fed the FC-diet; this was true in both wild-type mice and in the *Slc5a8*-null mice (Figure 3(b)). The abundance of *Bacteroidia*, which belongs to the *Bacteroidetes* phylum, decreased in wild-type mice when fed the FF-diet instead of the FC-diet, but the decrease was evident in *Slc5a8*-null mice independent of the fiber content in the diet when compared with FC-fed wild-type mice.

We performed indicator species analysis (ISA) to monitor bacterial species that are unique within a given Slc5a8 genotype and within a given dietary fiber condition. This analysis determines bacterial OTUs that are significantly associated with a given condition (*P* < 0.05) based on fidelity (exclusivity) and relative abundance of the organism. First, we compared wild-type mice fed the FC-diet with wild-type mice fed the FF-diet (Table 1).  Table 2 lists bacterial species that are unique to *Slc5a8*^−/−^ mice fed the FC-diet when compared with wild-type mice fed the FC-diet. Interestingly, the genera *Akkermansia* (phylum *Verrucomicrobia*) and *AF12* (phylum *Bacteroidetes*) were enriched in *Slc5a8*^−/−^ mice independent of dietary fiber content; in wild-type mice, the increase in the abundance of these genera was evident in animals fed the FF-diet compared with animals fed the FC-diet. *Desulfovibrio*, which belongs to the *Proteobacteria* phylum and also a well-known colitogenic bacterial genus, was present only in *Slc5a8*^−/−^ mice fed the FF-diet (Table 3).

### 3.3. Transcriptome Profile

To identify the global transcriptomic profiles associated with Slc5a8 genotype and the dietary fiber content, we performed RNAseq on colonic mucosal scrapings from the wild-type mice and *Slc5a8*-null mice fed either the FC-diet or the FF-diet. First, we identified differentially expressed genes (DEGs) using the cutoff set to a fold change of 2 and a *P* value of <0.05. In wild-type mice, 547 DEGs were identified between FC-diet and the FF-diet (434 upregulated genes and 113 downregulated genes). In *Slc5a8*^−/−^ mice, 143 DEGs were identified between FC-diet and the FF-diet (99 upregulated genes and 44 downregulated genes). Comparison between the two genotypes of Slc5a8 when fed the same diet showed 436 DEGs with the FC-diet and 267 DEGs with the FF-diet.

**Figure 4:**
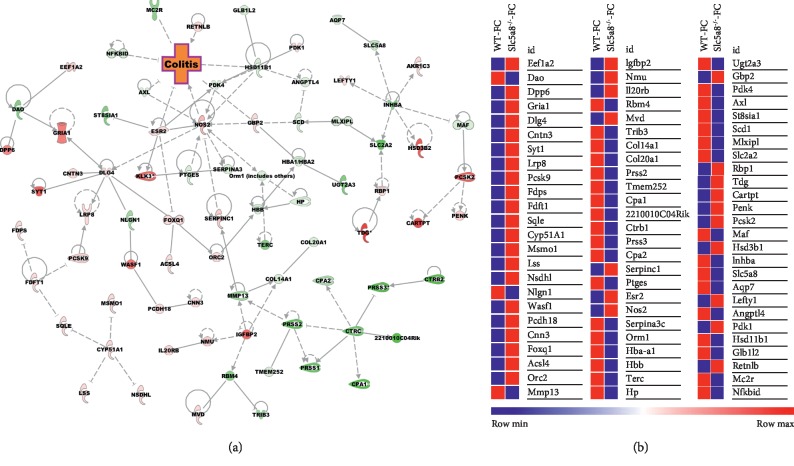
(a) Canonical pathway; (b) heat map of the genes related to the colitis signaling pathway in colonic epithelial cells obtained from wild-type (WT) and *Slc5a8*-null (KO) mice fed the fiber-containing diet (FC-diet). The significant pathway and DEGs were obtained via Ingenuity Pathway Analysis (IPA).

**Figure 5:**
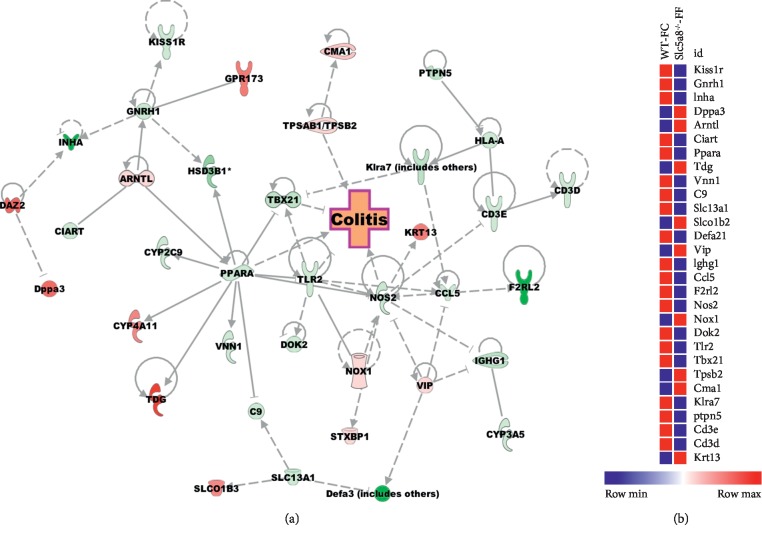
(a) Canonical pathway; (b) heat map of the genes related to the colitis signaling pathway in colonic epithelial cells obtained from wild-type (WT) and Slc5a8-null (KO) mice fed the fiber-free diet (FF-diet). The significant pathway and DEGs were obtained via Ingenuity Pathway Analysis (IPA).

**Figure 6:**
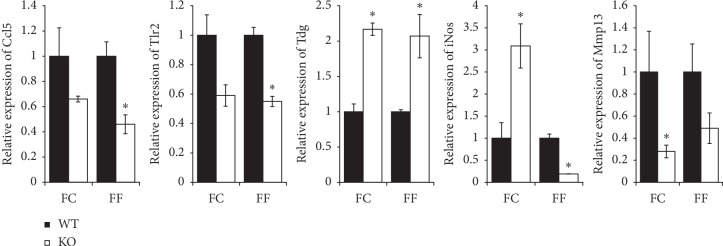
Quantitative PCR to confirm the RNAseq data in the four groups of mice (WT-FC, WT-FF, KO-FC, and KO-FF). The Student's two-tailed *t*-test was used to calculate statistical significance (*P* < 0.05; *n* = 6 mice/group). ^*∗*^*P* < 0.05 in KO mice when compared with WT mice when fed the respective diet.

We used Ingenuity Pathway Analysis (IPA) software to identify significant molecular pathways and functions which are different among the four groups. As the fiber content in the diet and the SCFA transporter Slc5a8 are principally associated with colonic inflammation, we focused on colitis while analyzing the transcriptome profiles by IPA. We found that deletion of Slc5a8 itself causes alterations of gene expression that is conducive for colitis, and deficiency of fiber in the diet exacerbates this phenomenon (Figures 4 and 5). To confirm the RNAseq data, we performed qRT-PCR for some of the differentially expressed genes such as Ccl5, Tlr2, Tdg, iNos, and Mmp13 (Figure 6). We observed decreased expression of Ccl5 and Tlr2 in *Slc5a8*^−/−^ mice than in wild-type mice irrespective of the dietary fiber content. Mmp13 gene expression also showed a similar trend. The expression of Tdg increased in both genotypes of mice irrespective of the fiber content of the diet. Interestingly, we found differential effects of the dietary fiber on iNos expression in wild-type mice and *Slc5a8*-null mice dictated by the fiber content in the diet. When fed the FC-diet, iNos expression increased in *Slc5a8*^−/−^ mice compared with wild-type mice, but the effect was opposite in the case of FF-diet. When fed this fiber-free diet, the *Slc5a8*-null mice showed decreased expression of iNos compared to wild-type mice.

### 3.4. Epithelial Barrier Layer Homeostasis and Repair

**Figure 7:**
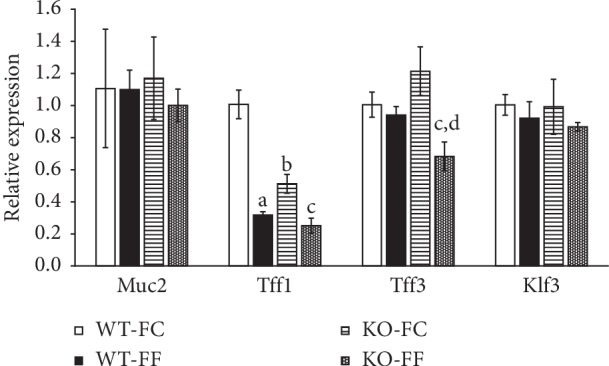
Quantitative PCR for four specific genes in colonic epithelial cells in wild-type mice (WT) and *Slc5a8*^−/−^ mice (KO) fed the fiber-containing diet (FC-diet) and the fiber-free diet (FF-diet). The Student's two-tailed *t*-test was used to calculate statistical significance (*n* = 6 mice/group). ^*∗*^*P* < 0.05 in WT-FF compared with WT-FC; $, *P* < 0.05 in KO-FC compared with WT-FC; @, *P* < 0.05 in KO-FF compared with WT-FF.

**Figure 8:**
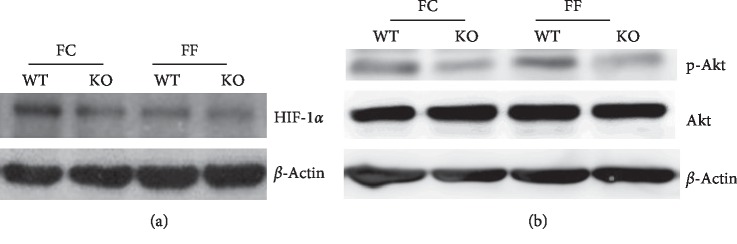
Western blot analysis of mucosal scrapings from colon in wild-type mice (WT) and *Slc5a8*^−/−^ mice fed with the fiber-containing diet (FC-diet) and the fiber-free diet (FF-diet). Images are representative 3 independent samples of mucosal scrapings from 3 individual mice.

To further analyze the mucous layer integrity, we examined the mucous building blocks Muc2a, mucosal repair factor Trefoil factor (Tff1), and Kruppel-like factor (Klf3) essential for barrier function. We observed significant downregulation of mucosal repair factor Tff1 in the absence of dietary fiber in wild-type mice and in Slc5a8-null mice. More importantly, deletion of Slc5a8 resulted in decreased expression of Tff1 in both dietary conditions (Figure 7). In contrast, expression of Tff3 decreased only in *Slc5a8*-null mice, that too only when fed the FF- diet. The expression of the other two genes (Muc2 and Klf3) did not change in any of the four groups. TFF3 is transcriptionally activated by PI3K/Akt signaling pathway. Therefore, we performed western blot analysis with mucosal scrapings from wild-type mice and *Slc5a8*^−/−^ mice fed either the FC-diet or the FF-diet (Figure 8). Akt phosphorylation decreased in *Slc5a8*^−/−^ mice compared with wild-type mice irrespective of the fiber content in the diet (Figure 8(a)). In wild-type mice, dietary fiber did not alter Akt phosphorylation. RNAseq analysis showed differential expression of Ccl5, iNos, and Mmp13. Hypoxia inducible factor-1*α* stabilization and activation induces the expression of these genes. Therefore, we monitored the levels of HIF-1*α* by western blot in the mucosal scrapings from the four groups of mice. HIF-1*α* levels decreased both in wild-type mice and in *Slc5a8*^−/−^ mice fed the fiber-free diet (Figure 8(b)). With the fiber-containing diet, HIF-1*α* levels decreased in *Slc5a8*-null mice compared with the wild-type mice.

## 4. Discussion

### 4.1. Influence of Dietary Fiber and Slc5a8 on Microbiota

Diet, gut microbiota, and host genetics are important factors for healthy living [25] and intestinal epithelium, primarily in the colon, is the site for interaction between diet, microbiota, and host [26]. Pathogenesis of IBD is driven by a multifactorial process, and one of the widely accepted causative factors is microbiota dysbiosis (i.e., alterations in the bacterial composition). Published reports have shown that dietary components influence the microbiota, and that microbiota in turn influences the epithelial barrier integrity and immunity in the host intestinal tract [27]. The integrity of the colonic epithelial barrier is critical for protection against IBD; it effectively prevents direct interaction between luminal bacteria and the host immune system, an obligatory process for successful symbiotic coexistence of the bacteria and the host. This does not mean that luminal bacteria do not communicate with the host immune system; they do but mostly via chemical messengers which can cross the intact epithelial barrier from the lumen to reach the immune cells present in the lamina propria [28–30]. Bacteria generate several metabolites using dietary fiber and proteins, which then elicit a broad spectrum of biological effects via activation of cell-surface receptors and nuclear receptors in epithelial and immune cells in the host [28–30]. In addition, some of these metabolites also work on pattern recognition receptors (PRR) such as toll-like receptors (TLRs) and nucleotide-binding oligomerization domain-like receptors (NLRs) in immune cells [27]. The intestinal epithelial layer consists of different cell types, including absorptive enterocytes/colonocytes responsible for nutrient absorption, secretory epithelial (Paneth and goblet cells) cells secrete antimicrobial peptides and mucins and hormone-secreting enteroendocrine cells. Paneth cells, present only in the small intestine secrete antimicrobial peptides (AMPs), whereas Goblet cells, a main subtype of intestinal epithelial cells present in the colon, are involved in the maintenance of barrier function via secretion of mucins, trefoil factors (Tff), and AMPs [26]. Mucins serve as a source of carbon and nitrogen for colonic bacteria when diet is deficient in fiber.

Previous studies from our laboratory have shown that the Na^+^-coupled high-affinity monocarboxylate transporter Slc5a8 functions as a tumor suppressor only when diet is deficient in fiber [15]. The current study was undertaken to investigate the interaction between dietary fiber and Slc5a8 in determining the composition of colonic bacteria and the gene expression pattern in colonic epithelium to understand why the biological consequences of Slc5a8 deletion become apparent only when the diet is deficient in fiber. SLC5A8 functions as a tumor suppressor not only in the colon but also in a wide variety of tissues [14]. In the colon, the principal driver of the tumor-suppressive function of this transporter is to mediate the Na^+^-coupled concentrative accumulation of the bacterial fermentation product propionates and butyrate in colonic epithelial cells, which are potent inhibitors of histone deacetylases. In noncolonic tissues, the transporter might function in the cellular accumulation of pyruvate, also an inhibitor of histone deacetylases [31–33]. There is also evidence that SLC5A8 might elicit its tumor-suppressive effects via a transport-independent mechanism involving interaction with survivin [34].

In the present study, we examined the influence of dietary fiber and its synergy with SLC5A8 on microbial composition in the colonic lumen and on the transcriptome profile of the colonic epithelium. Our studies clearly show that dietary fiber influences the composition of colonic bacteria. This is expected because different strains of bacteria prefer different carbohydrates as a carbon source for their metabolism and fermentation. Therefore, when the diet is deficient in fiber, some bacterial strains do not proliferate, whereas some others have a proliferative advantage under these conditions. This leads to significant differences in the strain composition of colonic bacteria. What is surprising, however, is the finding in the present study that the presence or absence of Slc5a8 in mouse colon also determines the composition of colonic microbiome. We found higher enrichment of colitogenic bacteria *Prevotella*, *Sutterella*, and *Erysipelotrichaceae* and decreased abundance of *Firmicutes Slc5a8*-null mice when fed the fiber-free diet compared to when fed the fiber-containing diet. In addition, the bacterial strains associated with disease remission in patients with ulcerative colitis, which include *Staphylococcaceae*, *Lactobacillaceae*, and *Coriobacteriaceae*, are also enriched in *Slc5a8*-null mice. The increased lactic acid-producing Bacteria (LAB) during active colitis has been previously reported [35]. It has also been reported that mice colonized with Phylum *Prevotella* were susceptible to experimental colitis [36]. Hamilton et al. [37] have reported that increased *Akkermansia* abundance in colon is associated with decreased thickness of the mucus layer and also with decreased number of mucin-producing goblet cells. Furthermore, *Akkermansia* spp., *Desulfovibrio* spp., and phylum *Prevotella* have more tendency to bind to inflamed colon compared to healthy colon [38, 39]. We found in our study that the mucolytic bacteria *Akkermansia* are in higher abundance in Slc5a8-null mice when fed the fiber-free diet. This could contribute to the thinning of the protective mucous layer in the colon, thus contributing to an increased risk of colitis under experimentally induced colonic inflammation. *Erysipelotrichaceae* have been associated with inflammatory diseases and metabolic disorders, both in humans and mice [40, 41]; this bacterial strain is also present in greater abundance in Slc5a8-null mice when fed the fiber-free diet. Similarly, the family of *Rikenellaceae*, which is also a mucin-degrading bacteria [42], is present abundantly in Slc5a8-null mice when fed either the fiber-containing diet or the fiber-free diet. During active colitis, overgrowth of *Bacteroides* and decreased abundance of *Firmicutes* have been reported [43]. *Enterococcus* abundance correlated with genetic mouse models of IBD and carcinoma [44]. The bacterial genera *Sutterella* belonging to the *Proteobacteria* phylum is known to possess a proinflammatory characteristic and is capable of adhering to intestinal epithelial cells [45]. These disease-associated bacterial genera are present in abundance in *Slc5a8*-null mice fed the fiber-free diet. The observed changes in bacterial composition in *Slc5a8*-null mice when fed the fiber-free diet, reflecting bacterial dysbiosis, and the increased abundance of disease-causing bacteria in these mice strongly suggest that the bacterial dysbiosis seen in these mice do contribute to the increased severity of inflammation in colon in experimental colitis as observed in our previous study [15].

### 4.2. Transcriptome Analysis

In the present study, we also analyzed the transcriptome of colonic epithelium to understand the impact of dietary fiber and Slc5a8 on the gene expression profile in these cells. Fiber deficiency altered the colonic mucosal transcriptome towards decreased epithelial repair and increased inflammation. HIF-1*α* is important for mucosal repair, and its signaling cascade has been shown to be protective against colitis [46]. HIF-1*α* expression is decreased in *Slc5a8*-null mice fed either the fiber-containing diet or the fiber-free diet. SCFAs generated by bacterial fermentation of dietary fiber in colonic lumen link Slc5a8 to HIF-1*α*; these bacterial metabolites are excellent energy substrates for colonic epithelium and are also known to promote stabilization of HIF-1*α* [47]. The same is true with Akt signaling. This pathway protects against colitis, but its activity is decreased in *Slc5a8*-null mice independent of fiber content in the diet.

Mucins are building blocks of mucus layer, and Tff1 and Tff3 are peptides secreted by the goblet cells that facilitate epithelial restitution and mucosal protection through binding with mucins [48]. In the present study, we found the expression of Tff1 and Tff3 to be downregulated in *Slc5a8*-null mice when fed the fiber-free diet. The expression of Tffs is under the control of Tlr2; we found the expression of Tlr2 to be suppressed in *Slc5a8*-null mice irrespective of the dietary fiber content. This explains why the expression of Tffs is decreased in the absence of Slc5a8. Stimulation of Tlr2 by microbiota increases the Tff3 expression via PI3K/AKT pathway in mice, whereas this effect is not seen in Tlr2-knockout mice [49]. Suppression of Tlr2 expression decreases regulatory immune cells and induces inflammation [50].

### 4.3. Conclusion

In summary, our studies provide new insight into the molecular mechanisms that underlie the proinflammatory phenotype in colon of *Slc5a8*-null mice under conditions of low-fiber diet. The combination of fiber deficiency and absence of Slc5a8 promote bacterial dysbiosis in colon that is conducive of a proinflammatory condition. In addition, the gene expression profile of the colonic epithelium is altered such that the signaling via HIF-1*α* and Akt is suppressed, and the secretion of the mucosal protective peptides Tffs by the goblet cells is compromised. Collectively, these changes in the luminal bacteria and in the biology of colonic epithelial layer promote a proinflammatory milieu, thus increasing the risk of colonic inflammation in Slc5a8-null mice when fed a diet deficient in fiber.

## Figures and Tables

**Table 1:** Indicative Species Analysis of wild-type mice fed with FF-diet when compared with FC-fed wild-type mice.

WT-FF			
Phyla	Family	Genera	*P* value
*Actinobacteria*	Bifidobacteriaceae	*Bifidobacterium*	0.005
*Actinobacteria*	Coriobacteriaceae	*Collinsella*	0.013
*Bacteroidetes*	Porphyromoadaceae	*Parabacteroides*	0.003
*Bacteroidetes*	Rikenellaceae	*AF12*	0.003
*Firmicutes*	Streptococcaceae	*Streptococcus*	0.003
*Firmicutes*	Erysipelotrichaceae	*Allobaculum*	0.003
*Firmicutes*	Erysipelotrichaceae	*Clostridium*	0.003
*Firmicutes*	Erysipelotrichaceae	*Coprobacillus*	0.003
*Verrucomicrobia*	Verrucomicrobiaceae	*Akkermansia*	0.003

**Table 2:** Indicative Species Analysis of *Slc5a8*^−/−^ mice fed FC-diet when compared with FC-fed wild-type mice.

*Slc5a8* ^−/−^-FC			
Phyla	Family	Genera	*P* value
*Actinobacteria*	Bifidobacteriaceae	*Bifidobacterium*	0.005
*Actinobacteria*	Coriobacteriaceae	*Collinsella*	0.013
*Bacteroidetes*	Porphyromoadaceae	*Parabacteroides*	0.003
*Bacteroidetes*	Rikenellaceae	*AF12*	0.003
*Firmicutes*	Erysipelotrichaceae	*Allobaculum*	0.003
*Firmicutes*	Erysipelotrichaceae	*Clostridium*	0.005
*Firmicutes*	Erysipelotrichaceae	*Coprobacillus*	0.003
*Firmicutes*	Streptococcaceae	*Streptococcus*	0.005
*Verrucomicrobia*	Verrucomicrobiaceae	*Akkermansia*	0.003

**Table 3:** Indicative Species Analysis of *Slc5a8*^−/−^ mice fed the FF-diet when compared with *Slc5a8*^−/−^ mice fed the FC-diet.

*Slc5a8* ^−/−^ *-*FF			
Phyla	Family	Genera	*P* value
*Actinobacteria*	Coriobacteriaceae	*Collinsella*	0.008
*Bacteroidetes*	Bacteroidaceae	*Bacteroides*	0.002
*Bacteroidetes*	Paraprevetellaceae	*Prevotella*	0.002
*Firmicutes*	Staphylococcaceae	*Staphylococcus*	0.033
*Firmicutes*	Erysipelotrichaceae	*RFN20*	0.002
*Firmicutes*	Streptococcaceae	*Lactococcus*	0.002
*Firmicutes*	Lactobacillaceae	*Lactobacillus*	0.002
*Firmicutes*	Enterococcaceae	*Enterococcus*	0.031
*Proteobacteria*	Alcaligenaceae	*Sutterella*	0.014
*Proteobacteria*	Desulfovibrionaceae	*Desulfovibrio*	0.036

## Data Availability

The RNA sequencing and 16S rRNA gene sequencing data used to support the findings of this study have been deposited in the NCBI-Sequence Read Archive (SRA) repository with bioidentification numbers as follows: PRJNA517543 and PRJNA515739.
